# Microfluidic 3D Bioprinting of Foamed Fibers with Controlled Micromorphology

**DOI:** 10.1021/acsami.4c22450

**Published:** 2025-02-18

**Authors:** Federico Serpe, Francesco Nalin, Maria Celeste Tirelli, Pasquale Posabella, Nehar Celikkin, Jakub Jaroszewicz, Wojciech Święszkowski, Andrea Barbetta, Efsun Şentürk, Carlo Massimo Casciola, Giancarlo Ruocco, Gianluca Cidonio, Chiara Scognamiglio, Marco Costantini

**Affiliations:** 1 Department of Chemistry, University of Rome “La Sapienza”, 00185 Rome, Italy; 2 Center for Life Nano- & Neuro-Science − CLN2S, Italian Institute of Technology (IIT), 00161 Rome, Italy; 3 Institute of Physical Chemistry, Polish Academy of Sciences, 01224 Warsaw, Poland; 4 Warsaw University of Technology, Faculty of Materials Science and Engineering, 02507 Warsaw, Poland; 5 Department of Mechanical and Aerospace Engineering (DIMA), University of Rome “La Sapienza”, 00184 Rome, Italy

**Keywords:** microfluidic, 3D bioprinting, foam, porous functionally graded materials, gradient, printhead

## Abstract

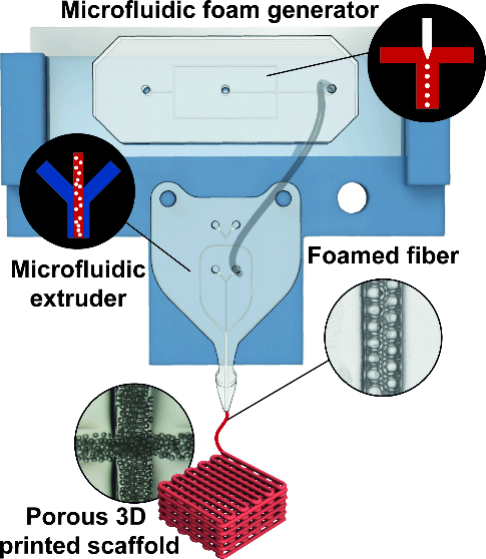

The synergistic integration of microfluidic technologies with additive manufacturing systems is advancing the development of innovative platforms to 3D bioprint scaffolds for tissue engineering with unparalleled biological relevance. Significant interest is growing in realizing porous functionally graded materials (pFGMs) that can resemble the hierarchical organization of porosity found in bone tissue. This study introduces a method for fabricating porous scaffolds based on the real-time generation of a liquid foam, which is gelled, forming porous fibers that are organized into structured matrixes using a 3D bioprinting system. The primary advantage of this approach is the possibility to adjust bubble size during printing dynamically, modifying the characteristics of the deposited foamed filaments online and in one step. As a result, locally-defined and tailor-made pores can be distributed in 3D structures with high spatial accuracy. Besides the mechanical and morphological characterization of diverse microarchitectures, we also explored the biocompatibility of the proposed approach by directly embedding osteosarcoma cells within the biomaterial. Results demonstrated the biocompatibility of the proposed methodology and revealed the influence of the interior microporosity on cell proliferation, highlighting the potential for creating tailored tissue microenvironments. The findings underscore the versatility of the presented 3D bioprinting system and its potential in fabricating biomimetic scaffolds with tailored morphological gradients, representing a substantial advancement in pFGM synthesis, with direct implications in regenerative medicine and tissue engineering.

## 1 Introduction

Lab-produced porous hydrogels have shown significant promise as cell culture and tissue engineering substrates. Their porous architecture supports cell adhesion and proliferation, facilitates the transport of nutrients and oxygen, aids in waste removal, and promotes the organization of cells into functional tissues.^1−3^ Over the years, substantial efforts have been made to improve the design of porous hydrogels, leading to the development of various production technologies.^4^ Conventional methods such as particle leaching,^5−7^ gas foaming,^8,9^ electrospinning,^10,11^ and freeze-drying^12,13^ have been widely employed in this field. However, these techniques often fail to precisely control key parameters such as pore diameter, polydispersity, and the spatial arrangement of interior pores.^14,15^ Insights from nature reveal that pores in natural materials are not randomly distributed but are organized in patterns optimized for specific functional properties, such as enhanced mechanical strength. Examples of such natural materials include bamboo, bone, and antlers.^16^

Recognizing the relationship between porous patterns and material properties has inspired the pursuit of advanced technologies capable of achieving precise control over pore structures.^17,18^ Among these advancements is the development of porous functionally graded materials (pFGMs)—materials designed with either discrete or continuous gradients in density and/or pore size, tailored to meet specific functional requirements. In this context, 3D printing technologies have driven significant progress in pFGMs fabrication. These technologies enable the precise design and fabrication of complex structures with unmatched repeatability and spatial accuracy.^19,20^ 3D printing has benefited the biomaterial science domain by fostering the development of novel biomaterials with high control over composition and architecture. In the former case, bioink composition is crucial to provide a matrix conducive to cell proliferation and maturation while maintaining high printing resolution. In the latter case, the production of scaffolds with desired 3D micro- and macro-morphology facilitates nutrient diffusion and waste removal while ensuring proper spatial organization to support intercellular signaling and neo-tissue formation.

Extrusion-based 3D printing has emerged as the most effective technique for fabricating multiscale porous scaffolds from a wide variety of biomaterial inks.^21−23^ This multiscale porosity is achieved by tailoring ink formulations or designing advanced microfluidic extruders. For instance, biphasic inks have been developed to create porosity within the interior of laid fibers. These inks incorporate dispersed solid, liquid, or gas phases that act as pore templates within a liquid matrix, as highlighted in recent reviews.^24^

Despite its utility, this approach often requires harmful chemicals, harsh cross-linking conditions, or purification steps, making it unsuitable for directly incorporating cells into the ink. A notable advancement addressing cytotoxicity is the development of aqueous two-phase emulsions (ATPS).^25−27^ ATPS systems rely on two polymeric aqueous solutions, free of surfactants, that phase-separate under specific conditions to form a water-in-water (W/W) emulsion. The latter represents a viable environment for cells suspended in the external phase, which undergoes gelation during the printing process when exposed to external stimuli. Despite ATPS-based bioinks have demonstrated significant potential in 3D printing applications,^25,27^ they have inherent limitations. More specifically, the porosity of the fibers is fixed and given by the droplet size of the internal phase: once the emulsion is formulated and loaded into the dispensing syringe, the pore size cannot be adjusted during the printing process. Beyond the pores within individual fibers, another porosity exists within the scaffold and is represented by the spaces between laid fibers. Varying these spaces—by modulating fiber thickness and spacing—is the only strategy to create graded porous structures. Another key drawback of ATPS bioinks is their short lifespan. As metastable systems, they degrade rapidly, restricting the time available for printing. Consequently, printability decreases, limiting the size and complexity of the attainable constructs. Additionally, ATPS bioinks generally exhibit low viscoelastic properties, making it difficult to produce complex geometries, such as overhanging structures.

An alternative approach to producing pFGMs involves the development of advanced extruders that function as printing heads. The integration of microfluidic technology with 3D bioprinting enables to dynamically adjust biomaterial porosity in real-time through flow control, resulting in the fabrication of hierarchically graded structures.^28^ For example, Marcotulli and co-workers demonstrated the 3D printing of porous structures with high control over the gradual variation of microporosity thanks to a microfluidic printing head that produces and extrudes an oil-in-water (O/W) emulsion. The latter was continuously generated and deposited within a suspending medium, which provided structural support and allowed for the spatial arrangement of the emulsion into complex architectures that would be difficult to achieve in air.^29^ This approach ensured stability by preventing emulsion destabilization during the printing process.

Foams are highly promising material inks for cell incorporation and for the fabrication of graded porous constructs. Indeed, including a gaseous phase does not compromise cell viability, as the gas is naturally and spontaneously eliminated. The employment of foams in direct 3D printing was already described in two recent articles,^30,31^ in which components such as gelatin methacrylate, poly(vinyl alcohol),^30^ and silk fibroin^31^ were included in the formulations thanks to their bioactivity and excellent foaming properties. Direct 3D printing of foams is attractive for its simplicity and avoidance of any potentially toxic surfactants, thus enabling the encapsulation of cells within the foam.^30^ In both cases, however, the foam generation and the printing process represented two distinct steps. Similarly to the case of ATPS, the preformation of foam does not allow for the real-time tuning of the foam properties (pore size, porosity, interconnectivity) across the scaffold volume, precluding the possibility of fabricating pFGMs in one step. Recent advancements have introduced innovative microfluidic designs that enable the dynamic regulation of droplet volumes using actuatable nozzles.^32^ By integrating these microfluidic technologies with 3D printing, researchers have synchronized bubble generation with the extrusion of gelatin-based aqueous solutions, achieving hierarchical porous structures with localized pore volume control across 3 orders of magnitude, from picoliters to nanoliters. Visser and colleagues recently proposed a method for printing polymer foams with real-time adjustable bubble size, volume fraction, and connectivity.^33^ This approach utilizes a core–shell microfluidic nozzle mounted on a 3-axis robotic stage to generate monodisperse liquid foam, which is solidified via photopolymerization on the flight from the extruder to the deposition stage. The technique enables the production of open-cell foams with air-filled bubbles or closed-cell foams with nitrogen-filled bubbles. In subsequent work, the ink composition was refined to use a surfactant-free formulation based on thiol–ene chemistries.^34^ Another innovative method, presented by Weber et al., incorporates a microfluidic device into a digital light processing (DLP) printer to generate size-controlled air bubbles within a GelMA aqueous solution.^35^ This system enables the printing of complex 2D and 3D geometries with varying porosities. Following fabrication, 3T3 fibroblasts are seeded onto the porous scaffolds, demonstrating high cell viability after 7 days across all bubble-size settings. Despite these advancements, just a few of the aforementioned methodologies have directly embedded cells in the foam to produce cell-laden constructs. The use of organic scaffold precursors or harsh cross-linking conditions has made it necessary to seed cells onto scaffolds post-fabrication. However, this approach often results in uneven cell colonization, even in scaffolds with regular and ordered porous structures.^36^

We propose a novel method for fabricating 3D hierarchical structures with locally defined porosity, combining a microfluidic device to generate liquid foam with an extruder that induces continuous gelation of the foamed fibers. These fibers are deposited in predesigned patterns using a 3D bioprinting system. This method enables printing single- and multiporosity structures with precise spatial control, creating 3D porous constructs featuring a hierarchical architecture and customizable intrafiber cavities. Unlike similar approaches,^33,37^ our method leverages hydrogel materials as bioink templates, enabling the efficient encapsulation of living cells. The functionality of the platform was evaluated using an osteosarcoma cell line (MG63) to explore the impact of complex 3D architectures on cellular behavior.

## 2 Materials and Methods

### 2.1 Microfluidic Chips Design and Fabrication

The strategy developed relies on using an extrusion bioprinter that integrates two customized microfluidic devices connected in series as the printing head. The first chip generates the precursor foam, while the second promotes physical crosslinking and extrudes the bubble-filled fiber. The bubble generator, fabricated through conventional photolithography, consists of a central channel delivering air and two lateral channels delivering the biomaterial ink. The channels meet at the flow-focusing junction, with a 50 × 50 μm^2^ section. A filter is placed at the air and the material ink inlet to prevent particles larger than 50 μm from passing, avoiding instabilities during the foaming process. On the two side channels just before the junction, two serpentines are designed to create high hydraulic resistance to guarantee a balanced flow from both sides. The final portion of the outlet channel is enlarged to allow the insertion of a 1 mm OD plastic tube connecting the two chips and reducing bubble velocity, enabling the visualization of bubbles’ flow in real-time. The extruder chip comprise a second flow-focusing junction that allow the liquid foam and the crosslinking solution to meet in a controlled manner to form and deposit the bubble-filled hydrogel fiber. In the case of bioprinting, the liquid foam is blended with a cell-laden biomaterial through a Tesla-inspired passive micromixer^38^ to obtain a homogeneous mixture of ink, cells and bubbles. Both extruder chips have been discussed and characterized previously.^200^

### 2.2 Biomaterial Ink Formulation

The developed biomaterial ink, referred to as LAG, comprised 0.5% w/v Laponite (XLG grade), 2% w/v alginate, 1.5% w/v gelatin previously used,^39^ with the addition of 0.125% v/v of Plantacare 2000. The composition and preparation methods were optimized to ensure stability and compatibility for cell encapsulation. Laponite (BYD, UK), a synthetic nanoclay, is particularly significant for bone tissue engineering due to its bioactive properties, which promote osteogenic differentiation and mimic the mineral phase of natural bone.^40,41^ To facilitate cell embedding, the prepared LAG ink was further sterilized under UV light for 30 min and maintained at 40 °C until cell resuspension. Including a surfactant (Plantacare 2000, BASF) is critical for stabilizing the foam during production. The surfactant was dissolved in distilled water and homogenized at room temperature before being combined with the powders.

The sheath fluid, consisting of 80% v/v glycerol in distilled water with 0.33 M CaCl_2_, facilitated fiber gelation, ensuring stable foam generation while supporting cell viability and distribution.

### 2.3 Foaming Process Characterization

To determine the characteristics of the produced foams, we observed the generation of air bubbles right after the flow-focusing junction of the bubble generator. We analyzed foams formed at a liquid phase flow rate of 25, 30, 35, and 40 μL/min. To carry out the measurements, the two chips were connected in series with a 5 cm plastic tube connecting the outlet of the foaming chip to the inlet of the extruder. The syringes controlling the biomaterial ink flow were driven by a syringe pump system (neMESYS low pressure, CETONI GmbH, Germany) while air pressure was finely regulated via a microfluidic controller (OB1 MK4, Elveflow, France). The air pressure was manually increased to 2000 mbar with pressure steps down to 30 mbar. To assess the formation of the liquid foam in real-time, an ultrahigh frame rate camera (FASTCAM SA5, Photron, Japan) was used to record videos through the Photron FASTCAM Viewer (PFV) software. The camera frame rate was set to 10000 and an intense light source was positioned at the bottom of the chip to provide sufficient illumination.

Following the acquisition, image analysis was performed on recorded video frames through ImageJ software to extract bubble production frequency, diameter and volume. To carry out dimensional analysis, we had to consider that bubbles would maintain a spherical shape only if their diameters were smaller than the channel height (50 μm). In comparison, they would flatten into a disc when the diameter exceeded 50 μm: in the first case, we considered the radius of the image as the radius of the sphere, while in the second case, we calculated the volume of a disk with a height of 50 μm and the radius calculated from the image. In both cases, we plotted the diameter of the sphere with the same volume as the bubble.

Once the bubble diameters have been measured for each flow rate-pressure condition, the distribution of bubble diameters was calculated only for the 35 μL/min flow rate condition, which will be later used for 3D printing. A 3D graph showing bubble size distribution at varying pressures was created by exploiting the *3D plot* function of Origin software (OriginLab, Massachusetts, USA). The polydispersity index (PDI) was calculated as^18^
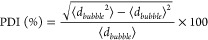
1To be considered monodisperse, the foam PDI should be below 5%. For values above 5%, the PDI is an indicator of polydispersity.^42^

### 2.4 Generation and Characterization of Foamed Fibers and 3D Deposition

For the formation and deposition of foamed fibers, the two chips were assembled onto a single holder to keep the foaming chip and the extruder in a stable vertical position during the printing phase. The foam coming out from the first chip was delivered to the inlet of the extruder through a 5 cm plastic tubing. Within the coaxial extruder, 80% v/v glycerol solution containing 0.33 M CaCl_2_ was used as sheath flow to shape the liquid foam into a solid filament and extrude it. The fiber was then deposited onto a glass substrate at a fixed speed of 12 mm/s in a simple pattern of straight lines to facilitate fiber diameter measurement. Air pressure was gradually varied from 700 to 1600 mbar. The images were acquired with an inverted microscope (ECLIPSE Ts2R, Nikon, Japan), and fiber diameter was calculated with the built-in ImageJ measuring tool.

### 2.5 Printing Foamed Scaffolds with Single and Multiple Porosities

3D-printed foamed scaffolds consist of well-organized fibers spatially arranged in a grid shape. Typically, 10 × 10 mm^2^ structures with a 1 mm interfiber distance were printed for all the foaming conditions. The 3D scaffolds were initially printed with a single foam density, maintained for the entire printing time. By tuning air pressure, we defined three different foam densities: *(i)* low-density foam (*low*), which includes less than 10% v/v of air volume, obtained by adjusting the air pressure between 700 and 800 mbar; *(ii)* medium-density foam (*medium*), where the air accounts for about 30% v/v of the biomaterial volume. In this case, the air pressure was maintained in the range of 900–1000 mbar; *(iii)* high-density foam (*high*), which contains above 50% v/v of air. In this case, the air pressures were set at values higher than 1400 mbar.

Then, complex scaffolds with hierarchical arrangements of different foam densities along the 3D structure were realized. Specifically, 3D scaffolds with step variation (*low-medium-high*, *i.e., step*), alternate distribution (*low-high-low-high, i.e., alternate*) and gradual increase or decrease (*gradient*) of the foam density were printed. The Elveflow Smart Interface (ESI) software was employed to design customized functions that automated air pressure variation. However, since fibers produced at different air pressure conditions vary widely in size, *Z* steps were adjusted accordingly. Specifically, for scaffolds with a progressive increase of foam density (*step* or *gradient*), the fiber size grows larger with each step. To accommodate these larger fibers, the *Z* step was increased incrementally by small amounts (+1% or +2% per layer). This ensures sufficient spacing between layers to prevent overlapping or structural deformation. For the *alternate* scaffolds, the *Z* step was varied heterogeneously as smaller and larger fibers alternated layer-by-layer. In addition, when transitioning between layers with different densities (e.g., in *step* or *alternate* scaffolds), the foam extrusion rate changes sharply due to pressure shifts. A customized external path was added between layers to stabilize the filament extrusion during these transitions and enhance printing performance, allowing the foam stream to normalize before deposition.

### 2.6 Mechanical Compression Tests

3D scaffolds with dimensions 10 × 10 mm^2^ with a fixed number of layers (i.e., 20) were analyzed through compression tests. After printing, samples were soaked in 0.33 M CaCl_2_ solution for 5 min to complete cross-linking and left in a 1% w/v solution of CaCl_2_ until the analysis. The scaffolds were removed from the liquid and gently dried with paper. All samples have been tested in triplicate. Sample heights were also extracted from compression measurements, considering the initial value on the *Z*-axis before starting the test.

The mechanical properties of the printed samples were evaluated in compression at room temperature using a dynamic mechanical analyzer (DMA Q800, TA Instruments, New Castle, DE, United States). A static force of 0.001 N was applied before testing. The tests were conducted at room temperature using a ramp strain with a strain rate of 5% min^–1^ until 60% compression. The recorded stress–strain curves were analyzed using Universal Analysis 2000 software (TA Instruments, New Castle, DE, United States). Young’s moduli were calculated from the slope of the initial quasi-linear region, fitting the derivative of the stress–strain curve with a horizontal line. The toughness of 3D printed scaffolds was derived as the integral of the stress–strain curve, which was quantified with a built-in function of GraphPad Prism 8 (GraphPad software Inc. La Jolla, CA).

### 2.7 Micro-CT Analysis

3D printed scaffolds were analyzed via micro-computed tomography (μCT) to disclose the internal porous microstructure. Before scanning, samples were chopped with a circular puncher of 4 mm diameter and completely dehydrated, substituting water with ethanol and then ethanol with hexamethyldisiloxane (HMDSO). Finally, HDMSO was replaced with sunflower oil to increase radiographic contrast. Each sample was soaked in a solution of (i) water/ethanol, (ii) ethanol/HMDSO and (iii) HMDSO/oil at 100:0, 80:20, 60:40, 40:60, 20:80, 0:100 ratios for 3 min. Images were acquired with a micro-CT platform (Xradia MicroXCT-400, Zeiss, Germany) using 40 kV voltage, 10 W power, and 0.18° rotation step in an angle interval of 184°. Room temperature (25 °C) and atmospheric pressure conditions were maintained for the whole acquisition. Acquired scans were processed with Imaris (Oxford Instruments, UK) software to obtain 3D reconstructions of the samples and 2D sections of the internal microstructure.

### 2.8 MG63 Cell Culture

MG63 osteosarcoma cell line (ATCC, USA, passage 14–17) were cultivated in DMEM high glucose cell culture medium (Sigma-Aldrich) supplemented with 10% fetal bovine serum (FBS non-USA origin, Sigma-Aldrich, UK), 1% (v/v) l-glutamine (Sigma-Aldrich, UK) and 1% (v/v) penicillin/streptomycin (Pen/Strep, Sigma-Aldrich, UK) and kept in culture at 37 °C with 5% of CO_2_ balanced-air, changing media every 3–4 days.

### 2.9 Material Optimization for Cell Encapsulation

For the bioprinting of cell-laden constructs, powders were dissolved in autoclaved, sterile-filtered distilled water after 30 min of UV sterilization. Plantacare 2000 was added at 1 and 0.5% v/v concentration, and cells were encapsulated in the biomaterial to check their viability after 24 h. As a control, the bioink without surfactant and a 2D culture were prepared.

Considering the low viability, we explored the possibility of foaming the ink at lower surfactant concentrations to increase cell survival. We confirmed the stable generation of bubbles within the microfluidic chip at 0.5%, 0.25% and 0.125% v/v of Plantacare concentration. Cell viability was then investigated for the minimum value tested (i.e., 0.125% v/v). Hence, this condition was used for all the experiments involving cell encapsulation.

### 2.10 Microfluidic Bioprinting of Cell-Laden Foams

To avoid a long contact time between cells and surfactant, the foamed ink—containing 0.125% v/v of Plantacare 2000—was mixed with the cell-laden bioink before gelation. For cell experiments, the foamed ink was prepared at *low* (20% v/v) or *high* (40% v/v) air fraction, while the bioink was laden with MG63 at a concentration of 15 × 10^6^ cells/ml. The two solutions were then mixed at a ratio of 2:1 (or 1:2), resulting in 4 different experimental conditions in which cell density, percentage of surfactant and porosity were modified as reported in Table 1. 3D printed samples were cultivated up to 21 days in DMEM high glucose supplemented with 10% FBS and 1% Pen/Strep and kept at 37 °C with 5% of CO_2_, changing media every 3–4 days.

**Table 1:** Description of the Different Experimental Conditions for Bioprinting Experiments

	Cell concentration	Surfactant concentration	Air fraction (initial)	Air fraction (final)
**Condition 1 (C1)**	10 × 10^6^ cells/mL	0.04%	20%	6.7%
**Condition 2 (C2)**	5 × 10^6^ cells/mL	0.08%	20%	13.3%
**Condition 3 (C3)**	10 × 10^6^ cells/mL	0.04%	40%	13.3%
**Condition 4 (C4)**	5 × 10^6^ cells/mL	0.08%	40%	26.7%

### 2.11 Viability of Cell-Laden Scaffolds

For viability assessment, cells were prelabeled with a lipophilic tracer (Vybrant DiD, λ_ex_ = 646 nm, λ_em_ = 663 nm, Sigma-Aldrich) following the manufacturer’s protocol before the experiment (day 0) and then stained on day 1, 7 and 21 using Calcein AM (λ_ex_ = 501 nm, λ_em_ = 521 nm, Invitrogen, Thermo Fisher Scientific). At each time point, cells were washed 3 times with HBSS, soaked in serum-free media with 0.6 μg/mL (w/v) of Calcein AM and placed in dark incubator for 1 h. Afterward, samples were washed 3 times with HBSS and imaged using a confocal microscope (Olympus FV1200) with a 488 nm laser. Cells that resulted positive for both Calcein and DiD were considered alive, while cells positive only for DiD were considered metabolically inactive (dead).

### 2.12 Biochemical Functionalization of the Bioink

Further encapsulation tests were carried out by completely replacing the nonfunctionalized alginate with RGD-immobilized alginate, obtained as previously reported.^43^ Moreover, microbial transglutaminase (μTG, Sigma-Aldrich) was added to the culture medium at 10 units/mL concentration to cross-link the gelatin enzymatically. μTG was removed the day after via simple media change. In this case, viability was assessed after 1 and 3 weeks following encapsulation.

### 2.13 Statistical Analysis

GraphPad Prism 8 was employed for statistical analysis. Obtained results were evaluated by one-way ANOVA with a Tukey’s multiple comparison test with a single pooled variance and considering significative difference if *P* < 0.05.

## 3 Results and Discussion

### 3.1 Experimental Setup and Bioink Foaming Characterization

**Figure 1:**
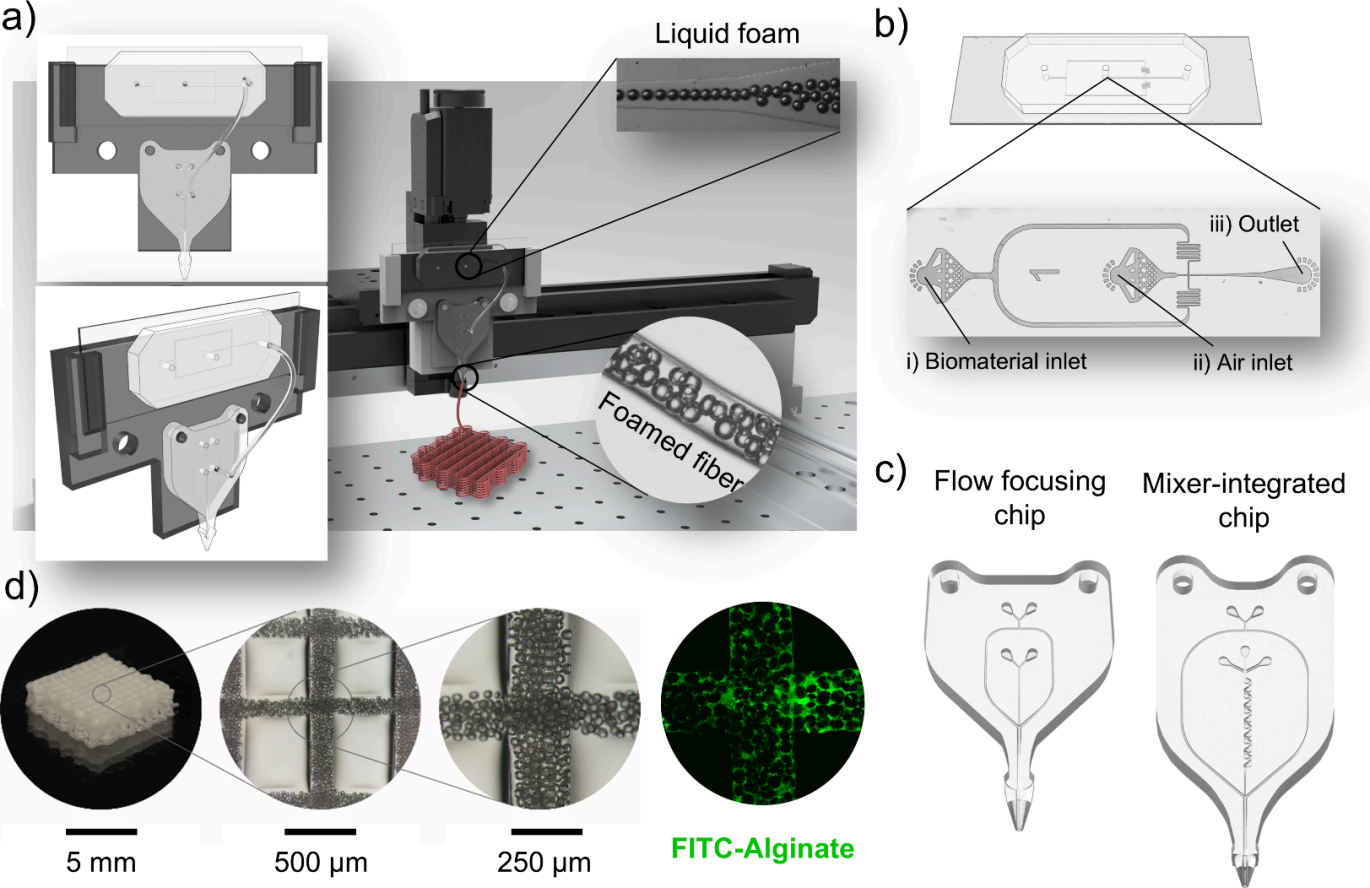
Set-up overview. (a) Rendering of the experimental setup with a close-up of the various components. In the background, the microfluidic printhead mounted on the bioprinter simulates the printing of a 3D scaffold (in red); on the left, front and lateral view of the printing head, composed of two microfluidic chips connected in series with a plastic tube. The two close-ups show the generation of the liquid foam in the first microchip and the formation of a solid bubble-filled fiber at the outlet of the second chip. (b) Rendering of the microfluidic bubble generator and enlarged image of internal microchannels. The chip is equipped with (i) an inlet for the biomaterial ink and (ii) an inlet for the air, both provided with a filtering structure and (iii) an outlet. (c) Rendering of the two extruders previously developed. The first chip contains a simple flow-focusing junction, while the second chip integrates a micromixer before the flow-focusing junction. (d) Macro- and micrographs of a 3D-printed foamed scaffold at different magnifications reveal the presence of tiny air bubbles embedded within the fibers. On the right, a fluorescence image of the printed foamed fibers containing FITC-labeled alginate confirms the presence of voids within the printed biomaterial ink.

The bioprinting setup comprises a 3-axis motorized machine that drives the microfluidic printing head in the 3D space. The latter is divided into two main components: the foam generator (upstream) and the extruder (downstream), from which the gelled, porous fiber comes out. Using different chips for the two units of the microfluidic printhead resulted in more stable and reproducible printing conditions. Moreover, the modularity of the printhead design facilitates its manufacturing, maintenance and use (Figure 1).

**Figure 2:**
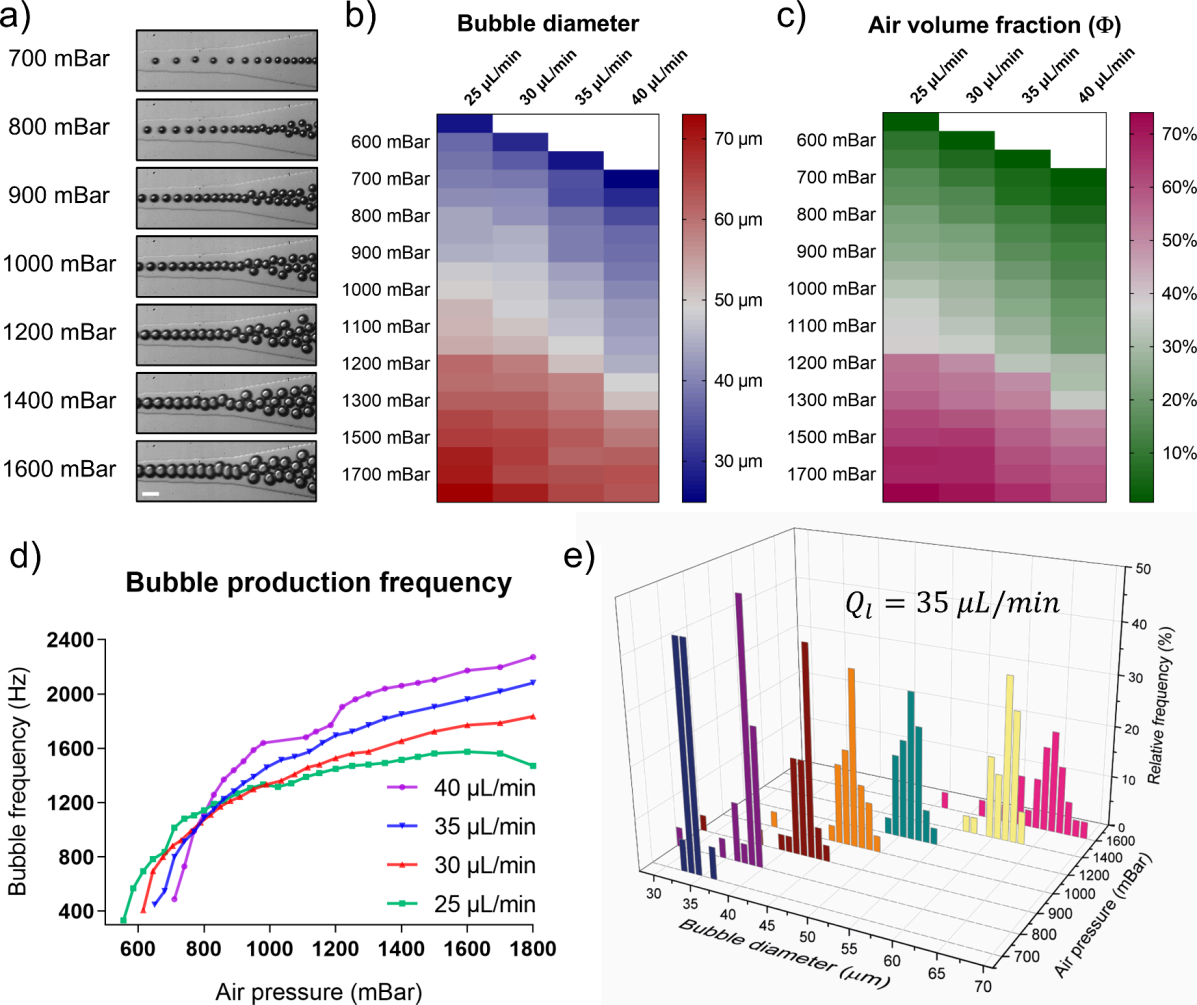
Characterization of the microfluidic foam. (a) Frames extracted from acquired videos reporting bubble formation fixing the flow rate to 35 μL/min and varying air pressure from 700 to 1600 mbar. The scale bar is 100 μm. (b) Heat map showing the mean bubble diameter estimated for different flow rates of the biomaterial ink (columns) and increasing air pressure values (rows). (c) Heat map showing the percentage of the air volume fraction calculated for different flow rates of the biomaterial ink (columns) and increasing air pressure values (rows). (d) Frequency of bubble production for 25, 30, 35, and 40 μL/min within the operational pressure range. The lines connecting dots are only to help the readers. (e) 3D histogram showing the distribution of bubble diameters for a fixed flow rate of 35 μL/min and pressure ranging from 700 to 1600 mbar. The occurrences are expressed as a percentage of the relative frequency. Results are expressed as mean ± SD of at least three replicates for each experiment.

To characterize the foam properties produced within the microfluidic chip in terms of bubble size, production frequency and volume, we acquired videos at 10000 fps during the foaming process at varying air pressure and flow rates (Figure 2a). As expected, the bubbles’ mean diameter—measured right after the flow focusing junction—was found to be a function of the applied pressure and liquid flow rate, spanning from 25 ± 1 to 73 ± 3 μm (Figure 2b). Such a range, despite covering only 50 μm, is appropriate in this context considering the characteristic size of laid fibers. The gas phase volume fraction was also quantified, resulting inversely proportional to the flow rate imposed (Figure 2c). Interestingly, above a critical value of air pressure, the air volume fraction did not increase further, reaching a plateau that resulted higher for lower flow rates. More specifically, air fraction reached 60% and 74% at 40 μL/min and 25 μL/min, respectively, at a pressure of 1800 mbar. This demonstrated that the less the liquid injected through the flow-focusing junction, the higher the volume occupied by the gas. In fact, at constant air pressure, the bubble pinching time increases when the liquid flow rate is reduced, allowing each bubble to grow larger before detaching from the junction.

Afterwards, we determined bubbling frequency—an index of the bubble production rate—as a function of the applied air pressure for each tested flow rate (Figure 2d). The minimum pressure required for bubbling increased from 555 mbar to 710 mbar when the flow rate changed from 25 μL/min to 40 μL/min. Indeed, the increasing shear force exerted by the liquid demanded greater gas pressure to start the generation of microbubbles.^44^ The behavior at different flow rates was similar, presenting a nonlinear trend of the frequency of bubble production as a function of the pressure. The dependence of the production frequency on the flow rate was already found to be directly proportional in a previous study.^45^ It can be explained by the fact that the shear forces associated with a higher flow rate of the continuous phase resulted in the formation of smaller bubbles but with higher frequency. More specifically, the maximum frequency achieved when the biomaterial ink was flown at 40 μL/min was 2.3 kHz. This value falls within the same order of magnitude of frequency reported in similar studies.^32^

The polydispersity of produced bubbles, which measures the homogeneity of bubble sizes, is another crucial aspect to be considered, as it can indirectly measure the stability of the microfluidic foaming process. Indeed, one of the major advantages of microfluidic technology is the sustained generation of dispersions with a low degree of polydispersity and with high throughput. The 3D histogram in Figure 2e provides a comprehensive view of the polydispersity of bubbles generated at different pressure conditions with a fixed flow rate of 35 μL/min. For each condition, the PDI was calculated (Supporting Information, Table S1). We observed that the degree of polydispersity was low for all the pressure conditions analyzed, specifically less than 10%. In a few cases, the foam can be considered monodisperse (i.e., PDI < 5%), but no specific trend was found as pressure varied. Estimated PDI values were in line with the ones reported in the literature for similar biopolymeric foams.^17,18,32^

### 3.2 Foamed Fibers Diameter Is Modulated with Air Pressure

**Figure 3:**
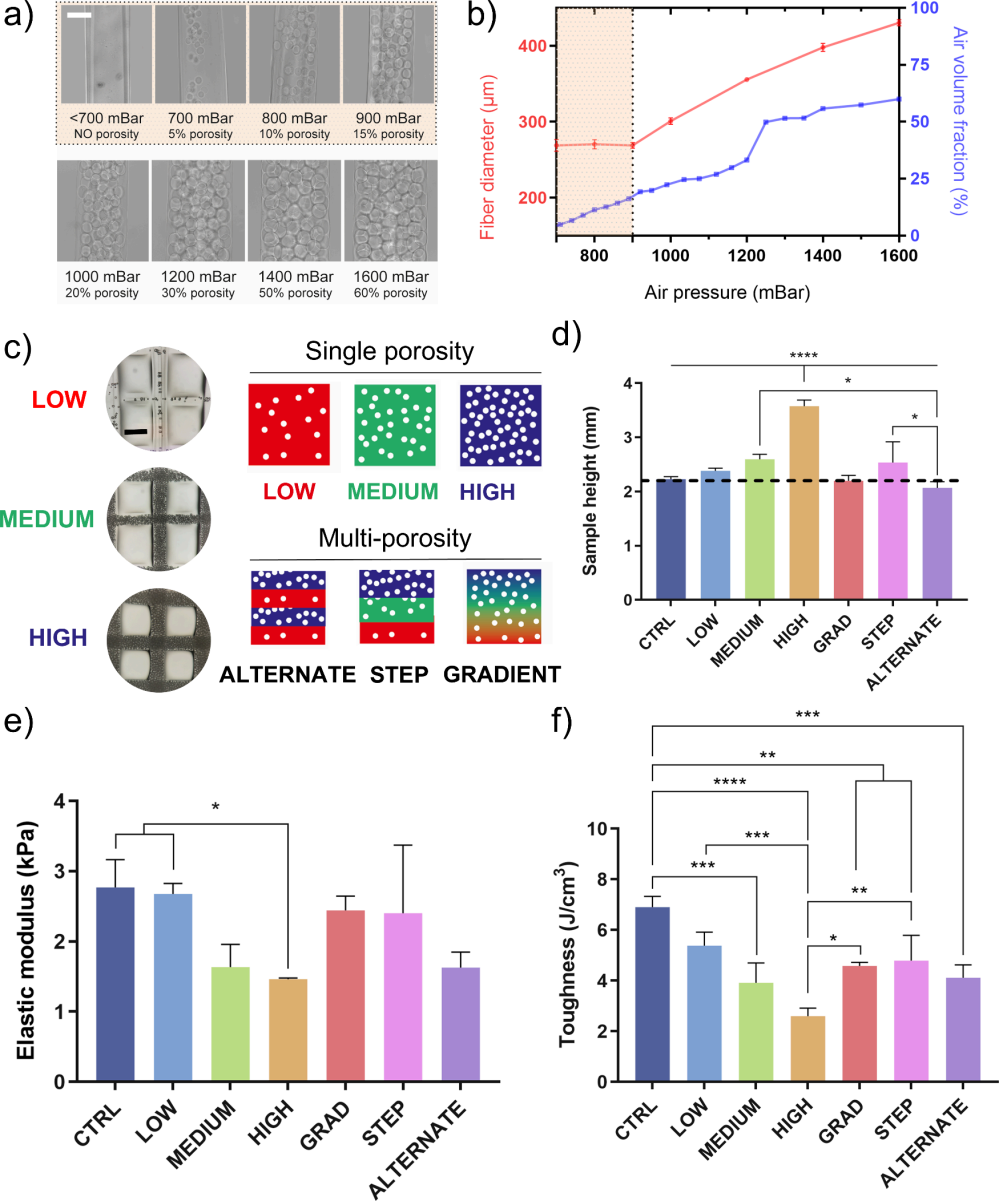
Diameter of foamed fibers and foamed scaffolds properties. a) Microscope images of deposited foamed fibers at different air pressures. The images encased in the colored box represent all the pressure values, which do not cause an increase in fiber diameter. The scale bar is 100 μm. b) Graph showing the diameter of foam-filled fibers (red line) and the correspondent volume fraction (blue line) as a function of the air pressure injected with a fixed flow rate of 35 μL/min. The portion of the graph with colored background represents the range of pressure values that keep the fiber diameter constant. c) On the left, a close-up of 3D lattices realized with foamed fibers, showing an increase in bubble size and number for different air pressure conditions. On the right, the schematization of the different types of 3D-printed scaffolds. While single-porosity scaffolds present a constant porosity along the 3D structure, multi-porosity constructs vary their internal porosity in 3D with predetermined patterns. d) Heights of the single- and multi-porosity 3D printed scaffolds. The dashed line represents the height of the control samples (non-foamed). e) Elastic modulus of 3D printed porous constructs. f) Toughness of 3D printed porous constructs. Statistical significance was calculated via one-way ANOVA. Results are expressed as mean ± SD of at least three replicates for each experiment, *p*<*0.05, **p*<*0.01, ***p*<*0.001, ****p*<*0.0001.

In vision of the printing step, where the fiber extrusion velocity must match the printing speed, we set the flow rate for the biomaterial ink and cross-linking solution at 35 μL/min and 30 μL/min, respectively. This flow rate combination has been experimentally proven to be well paired with the feed rate selected for 3D printing (FR = 12 mm/s). Following the foam generation, the foamed bioink is sent to the extruder chip to be shaped into fibers. The foam and cross-linking solution containing calcium ions flow into two distinct, coaxially aligned channels. Upon contact with the calcium ion solution at the extruder outlet, the foam containing alginate in the aqueous phase experiences immediate gelation, thereby inhibiting any degradation of the foam as air bubbles remain trapped within the gelled fiber. At constant flow rate, the foam density could be adjusted by varying the air pressure from 700 to 1600 mbar, resulting—at the end of the process—in porous fibers with different pore sizes, air fraction and tunable diameter (Figure 3a).

The diameters of the porous fibers were measured as a function of the applied air pressure (Figure 3b). Until a certain threshold value, the fibers maintained a constant diameter and the air bubbles appeared well-confined inside the filament. Bubbles started to blow the fiber when the pressure overcame the threshold, increasing its diameter, as also reported previously.^33^ At 35 μL/min, the diameter of the fiber measured 269 ± 8 μm until the threshold pressure of 900 mbar, corresponding to about 20% v/v of air volume fraction. After this value, fiber diameter grows almost linearly until reaching 430 ± 4 μm at the highest pressure tested. In this condition, the fiber appeared packed with bubbles neatly arranged and separated by thin walls (Figure 3a). The air volume fraction follows an analogous trend, exhibiting an increase proportional to the imposed pressure (Figure 3b). The linear behavior was maintained until 1600 mbar, corresponding to about 60% of the air volume fraction. The presence of an upper shift of the curve at around 1200 mbar could derive from the approximation we made for calculating the volumes of bubbles (which change from spheres to disc-like shapes). Indeed, it must be considered that bubbles were not shaped into disks with sharp edges, but instead, they deformed into flattened spheres. Therefore, their volume can be easily overestimated.

### 3.3 Fabrication of 3D Scaffolds with Constant and Hierarchical Porosity

The described foaming system was then coupled with a custom-made 3D printing set up to control the spatial deposition of foamed fibers (Supporting Information, Video S1). We reported the successful printing of 10 × 10 mm^2^ well-organized 3D structures with variable porosity densities. According to the conditions previously described in section 2.5, single and multiple levels of porosities were patterned in the axial direction of 3D constructs (Figure 3c), confirming the possibility of realizing hierarchical 3D structures with tailored porosities in a reliable and repeatable manner. As already pointed out, porous fibers modify their diameters in a wide range, resulting in the fabrication of 3D constructs with variable heights (Figure 3d). In particular, 3D scaffolds with *high* porosity extended their height significantly, reaching 3.6 mm, resulting 37% taller than the control scaffolds.

### 3.4 Mechanical Characterization of Single- and Multiporosity Scaffolds

The mechanical properties of 3D-printed scaffolds with varying porosity degrees and patterns (section 2.5) were evaluated through compression tests and compared against solid fiber 3D-printed scaffolds (*control*, Supporting Information, Figure S1a,b,c). Notably, the grid-style fiber deposition introduced macroporosity into the final constructs, significantly influencing their mechanical behavior. However, this effect remained consistent across all samples due to the uniform grid patterns and material composition, enabling a reliable comparison of mechanical performances. Indeed, as reported in the stress–strain curves of the samples with constant porosity, an appreciable reduction in magnitude was observed as porosity increased (Supporting Information, Figure S1e). To comprehensively assess the mechanical properties of the foamed scaffold, we calculated both the elastic modulus and toughness. The latter, defined as the area under the stress–strain curve (AUC), is an important metric for porous materials, reflecting their capacity to absorb mechanical or acoustic energy. Since toughness depends on micro- and nanoscale properties, it provides valuable insights into how variations in microbubble content influence the mechanical behavior of the scaffold.^46^

As shown in Figure 3e, Young’s modulus of scaffolds with uniform porosity decreased as fiber foaming increased. Specifically, the mean Young’s moduli of porous constructs showed reductions of 3% (*low*), 41% (*medium*) and 47% (*high*) relative to the average value obtained from control scaffolds. A significant difference was observed between *high* and *control* samples and *high* and *low* samples, whereas no significant variation was found between *medium* and *high* porosity samples. Conversely, toughness demonstrated greater sensitivity to slight changes in porosity (Figure 3f). *Low*, *medium* and *high* constructs exhibited mean toughness reductions of 22%, 43% and 62%, respectively, compared to control scaffolds. This analysis highlighted a strong dependence of mechanical behavior on air volume fraction, confirming that toughness was more sensitive to variations in internal microporosity compared to elasticity, which was predominantly biased by the presence of macroporosity.

In contrast to uniformly foamed structures, where stiffness is primarily determined by total porosity, the stiffness of layered foamed structures is influenced by the spatial distribution of micropores within the struts.^47^ For 3D-printed foams with varying foam densities, differences in elastic modulus and toughness between samples were less pronounced and generally fell within the average values estimated for *high* and *low* porosity samples. As visible from the graph in Supporting Information, Figure S1f, these variations were undetectable through compression tests, probably due to the low total polymer content of the biomaterial (4% w/v).

### 3.5 Morphological Characterization of Single- and Multiporosity Scaffolds

**Figure 4:**
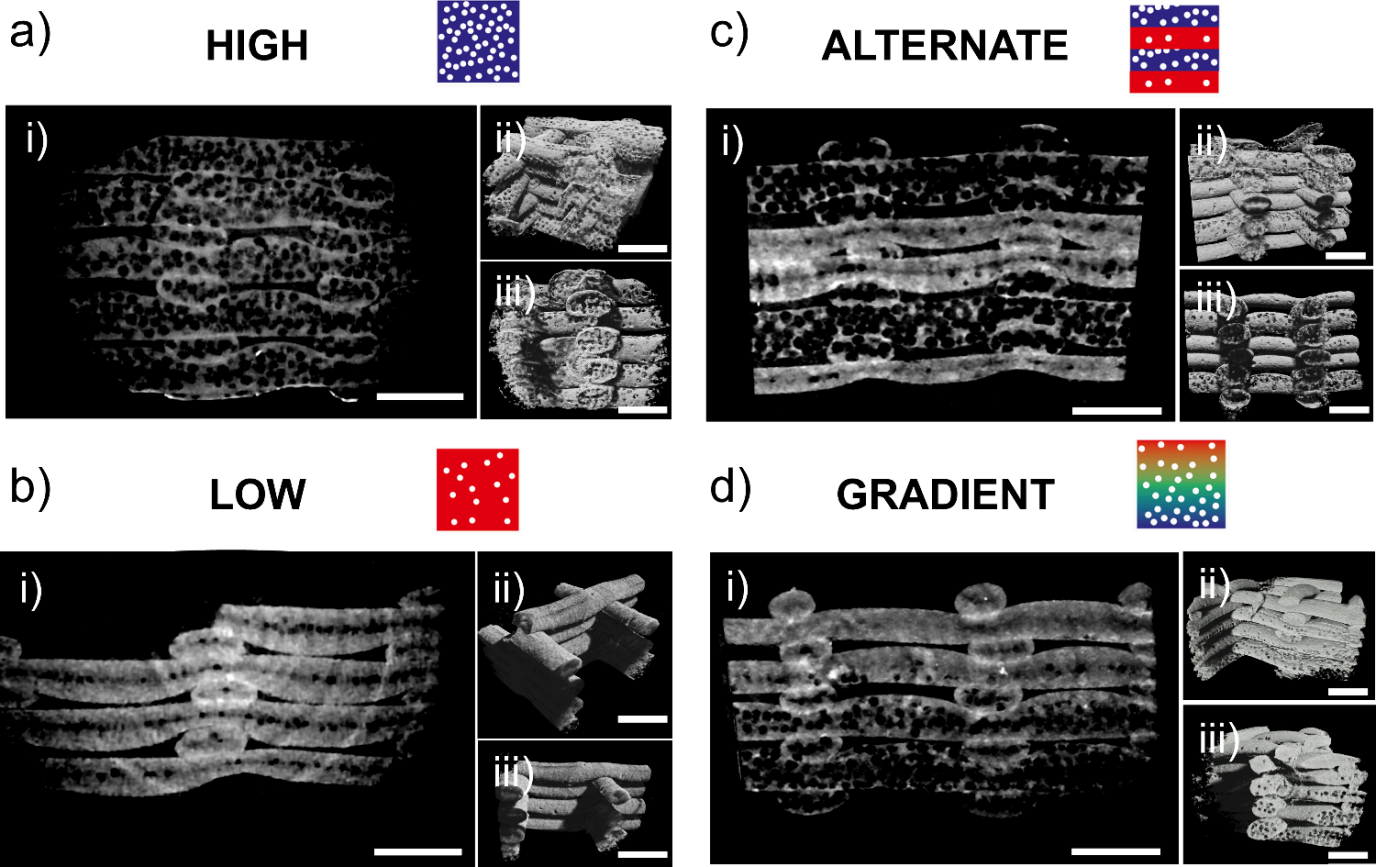
μCT scans of single- and multiporosity constructs. (a) Tomographic analysis of high porosity scaffolds. (i) 2D projection of an axial cross-section to visualize internal fiber porosity. On the right, reconstructions of 3D printed constructs were observed from a (ii) 45° and (iii) 90° angle. (b) Tomographic analysis of low porosity scaffolds. (i) 2D projection of an axial cross-section to visualize internal fiber porosity. On the right, reconstructions of 3D printed constructs were observed from a (ii) 45° and (iii) 90° angle. (c) Tomographic analysis of scaffolds with alternate porosity. (i) 2D projection of an axial cross-section to visualize internal fiber porosity. On the right, reconstructions of 3D printed constructs were observed from a (ii) 45° and (iii) 90° angle. (d) Tomographic analysis of scaffolds with gradual porosity variation. (i) 2D projection of an axial cross-section to visualize internal fiber porosity. On the right, reconstructions of 3D printed constructs were observed from a (ii) 45° and (iii) 90° angle. All scale bars are 500 μm.

We performed a micro tomographic analysis of the 3D-printed samples to visualize better the porosity generated within fibers. Both monodisperse samples with higher and lower porosity (*high* and *low*, Figure 4a,b) and multi-porosity scaffolds (*alternate* and *gradient*, Figure 4c,d) were scanned and reconstructed in 3D. For each sample, a 2D projection of a 7 μm thickness axial section was included to clearly show the presence of air voids and observe their dimension and spatial distribution. In the processed images, air bubbles trapped in the fibers appeared as black circles while the lighter signal represents the biomaterial matrix.

In monodisperse 3D scaffolds, a clear difference is observed in two representative degrees of porosity (i.e., *low* and *high*). As expected, internal voids appeared larger in number and size when porosity was increased. Regarding spatial distribution, we can notice how pores were distributed more homogeneously in *high* porosity samples with respect to *low* porosity ones, in which air bubbles tend to accumulate along the fiber axis. In 3D reconstructions, low porosity fibers have a solid-like appearance, while high porosity fibers exhibit an interconnected porous texture. The latter may result from the formation of extremely thin walls that cannot be discriminated through μCT.

For multilayered 3D scaffolds, μCT analysis confirmed the successful fabrication of structures with hierarchical pore distribution. Both *alternate* (Figure 4c) and *gradient* (Figure 4d) scaffolds demonstrated the anticipated spatial organization of porosity, as evident from the 2D sections. In *alternate* scaffolds, low-porosity layers were distinctly separated from high-porosity layers, with a sharp transition between them. Notably, the 3D structure remained stable despite the sharp flux variations caused by sudden increases in gas pressure. In contrast, *gradient* scaffolds exhibited a smooth reduction in air fraction along the axial direction, corresponding to the gradual decrease in applied air pressure. Although the continuous variation in air pressure influenced extrusion velocity and filament dimensions, the deposition was unaffected. Both 2D projections and 3D reconstructions revealed that gradient scaffolds were compact and well-organized, validating the successful printing of a porous, graded 3D structure. However, at higher porosity levels, the signal from the thin walls was notably weaker, leading to a void appearance on the external fiber surfaces in the 3D reconstructions.

### 3.6 Material Optimization for Bioprinting

Before proceeding with cell encapsulation in foamed constructs, we first evaluated the biocompatibility of the biomaterial ink. Although the individual precursors in the formulation are known for their biocompatibility, including a surfactant raised specific concerns. Determining the optimal surfactant concentration to balance bubble stability during foam generation and maintain cell viability was crucial.

The encapsulation of MG63 cells within the bioink at varying surfactant concentrations (0.5% and 1% v/v) was evaluated compared to 2D cultures and cells embedded in a surfactant-free biomaterial (Supporting Information, Figure S2a). After 24 h, we assessed the impact of the surfactant on cell viability. A Plantacare 2000 concentration of 1% v/v not only resulted in complete cell death but also caused significant damage, as indicated by the rupture of cell membranes and the loss of nuclear staining performed just before encapsulation. Also, after halving the surfactant concentration to 0.5% v/v, all encapsulated cells were nonviable. These findings suggest that Plantacare 2000 concentrations equal to or greater than 0.5% v/v do not provide a viable environment for encapsulated cells.

Given the essential role of the surfactant in foam generation, we investigated the effect of lower concentrations of Plantacare 2000 on the foaming process. Serial dilutions of the initial concentration (0.5%, 0.25%, and 0.125% v/v) were tested in the biomaterial ink to assess potential variations in foaming ability. Remarkably, all tested dilutions maintained consistent performance during the bubbling phase, demonstrating that similar experimental results could be achieved with reduced Plantacare concentrations (Supporting Information, Figure S2b).

We selected the lowest concentration tested (0.125% v/v) for bioprinting experiments to minimize the impact on cellular response. Furthermore, we reduced the risk of material-related cytotoxicity by minimizing the contact time between cells and the surfactant. This was achieved by blending the surfactant-free, cell-laden ink with the foamed material immediately before extrusion using a passive microfluidic mixer.^200^ This approach reduced the final surfactant concentration to below 0.1% v/v for each condition (Supporting Information, Table S1, Figure S2c).

### 3.7 Viability and Functionality of MG63-Laden Scaffolds

To fabricate cell-laden 3D foamed constructs via 3D bioprinting, an osteosarcoma cell line (MG63) was embedded in the liquid foam before gelation. The micromixer homogenized the foam and the cell-laden biomaterial before cross-linking and deposition of the filament. Cell viability was assessed on days 1, 7, and 21 using calcein staining and Vybrant DiD prelabeling.

Among the four printing conditions tested (Supporting Information, Table S1, Figure S3a), only condition C1 supported cell viability after 21 days of culture while, for all the other conditions, cells were found dead already 1 week after encapsulation. Specifically, C1 featured the highest cell density (10 × 10^6^ cells/mL) and the lowest surfactant concentration, creating a favorable environment for cell survival (Supporting Information, Figure S3b). While C3 also had the same cell density as C1, the higher bubble content entailed an increase in the bioink-air interface, where surfactant adsorption occurs. This phenomenon exposed more cells to surfactant, reducing their viability compared to C1. For the remaining conditions (C2 and C4), the bioink formulations were less cell-dense, rendering survival conditions more challenging in both cases. These results underscore the importance of optimizing cell density and surfactant concentration for successful long-term cell viability in 3D foamed constructs.

**Figure 5:**
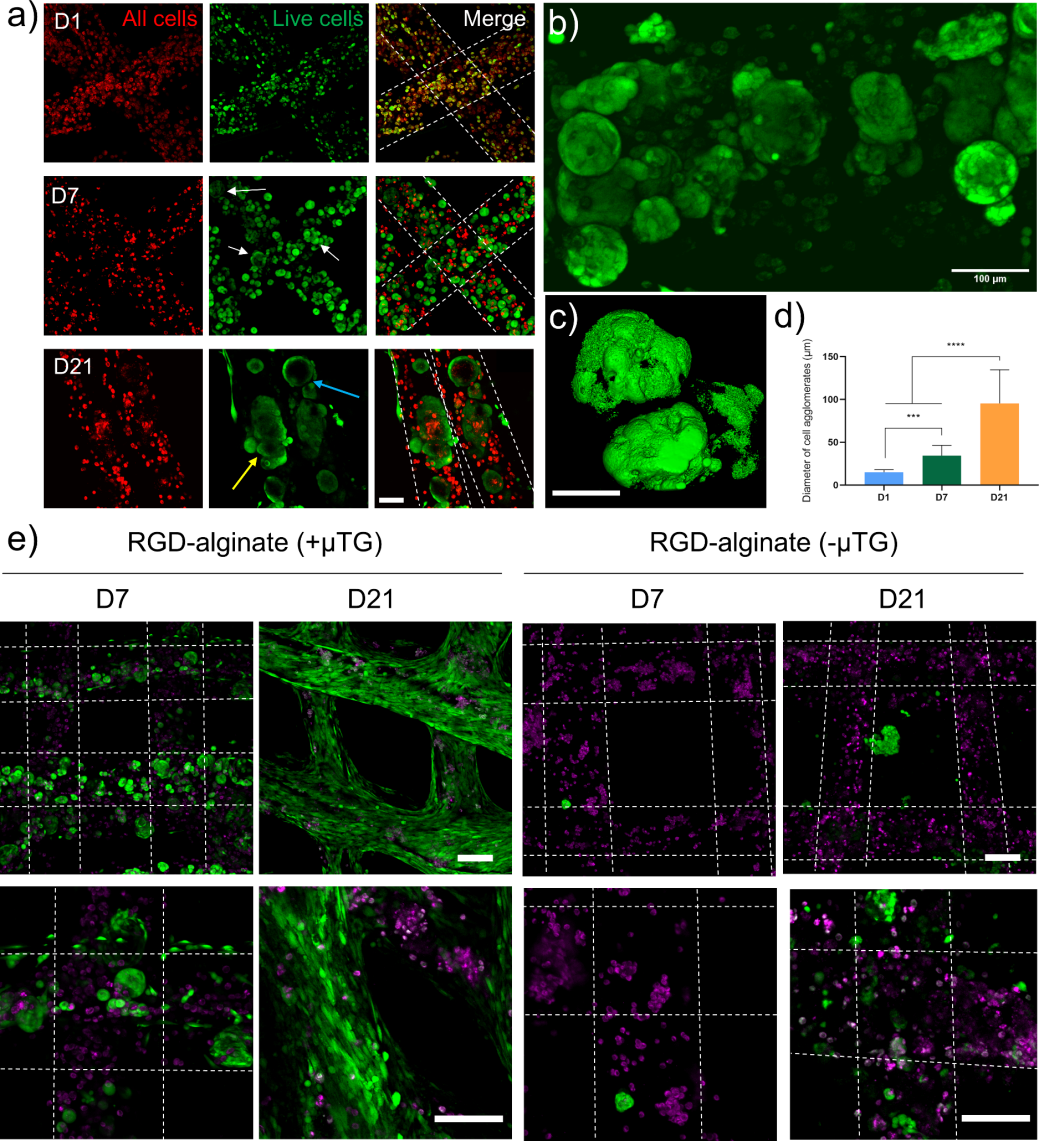
Viability staining of MG63 cells encapsulated in the foamed biomaterial ink. (a) Observation of cellular viability during 3 weeks of culture in condition C1. Bioprinted MG63 cells calcein staining on days 1, 7, and 21. Cells were prelabeled before encapsulation (red) and were marked green only if metabolically active. White arrows indicate clusters of 30–70 μm in diameter forming on day 7, while yellow and light blue arrows indicate the presence of massive clusters (70–120 μm in diameter) on day 21. These agglomerates appear elongated (yellow arrow) or perfectly round (light blue arrow), whether they are formed due to coalesced or single bubbles, respectively. The scale bar is 100 μm. (b) An enlarged image showing the formation of cellular clusters of different shapes and dimensions on day 21. (c) 3D reconstruction of a round cluster of bubbles to show the absence of cells in the central area. (d) Evaluation of the mean diameter of cellular aggregates formed within foamed fibers. (e) Comparison of the calcein staining performed after 1 and 3 weeks of culture in samples containing RGD-immobilized alginate that were treated or not with μTG shows a difference in cell proliferation after 1 and 3 weeks. Scale bars are all 200 μm. Statistical significance was calculated via one-way ANOVA. Results are expressed as mean ± SD of at least three replicates for each experiment, ***p < 0.001, ****p < 0.0001.

Being the only condition that remained viable for 3 weeks, images acquired on days 1, 7, and 21 for C1 are presented in Figure 5a. Calcein is a green-fluorescent dye that not only stains living cells but also provides insights into cellular morphology as it diffuses and accumulates in the cytoplasm. Over the 21-day culture period, significant changes in cell morphology were observed. On day 1, encapsulated cells were homogeneously distributed within the material, with a few bubbles interspersed throughout (resulting in an ultimate porosity of ∼7%). The prelabeled cells (red) were mostly viable, as evidenced by the colocalization of calcein (green) and the nuclear dye signals.

By day 7, cells began to cluster into aggregates 20–40 μm in diameter (white arrows). However, the nuclear prelabeling dye and calcein signals ceased to colocalize, suggesting that the cells had proliferated and the initially fluorescently stained cells died. On day 21, cell morphology underwent drastic changes, forming larger agglomerates ranging from 70 to 120 μm in size. Two distinct morphologies were observed: elongated clusters (yellow arrow) and circular clusters (light blue arrow). These configurations likely resulted from cell migration into voids left by air bubbles. Spherical clusters were probably formed by cells colonizing individual bubbles, while elongated clusters may have formed either from merging spherical aggregates or cells colonizing interconnected pores. Conversely, in the control (i.e., non-foamed) scaffolds, we observed living cells mostly growing on the external surface of the fibers in a quasi-2D arrangement, while cells located inside the fibers were found dead (Supporting Information, Video S2, Figure S4a).

A closer examination of the spherical agglomerates on day 21 (Figure 5b) revealed, at higher magnifications, both perfectly spherical clusters and elongated formations. 3D reconstructions (Figure 5c) demonstrated that cells grew along the internal surfaces of pores rather than forming compact spheroids (Supporting Information, Video S3). An analysis of the dimensions of the spherical clusters (Figure 5d) further confirmed significant growth over time. After 7 days, distinct mini aggregates averaged 34 ± 12 μm in diameter. By the third week, sparse cell packings were replaced by large clusters with considerable size variation, averaging 95 ± 39 μm. The fluorescence signal from prelabeling diminished over time, with no detectable signal in individual fibers and only faint signals within cell clusters by day 21.

Further encapsulation tests of MG63 cells were conducted following the specifications of C1, but with alginate replaced with a chemically-modified version, which incorporated covalently attached arginyl-glycyl-aspartic acid (RGD) motifs. Functionalizing biomaterials with RGD motifs has been demonstrated to enhance cell-biomaterial adhesion and promote the migration of encapsulated cells significantly.^48^ This is particularly important for alginate, which lacks intrinsic cell-binding sites; RGD motifs enabled cells to adhere to the 3D scaffold and facilitated their migration and network formation.^49−52^

Additionally, a subset of the RGD-modified alginate samples was treated with microbial transglutaminase (μTG), an enzyme that covalently bonds glutamine and lysine residues in gelatin. This treatment improved the biomaterial long-term stability, slowing gelatin degradation/dissolution during culture. Calcein staining was performed after 1 and 3 weeks to assess cell viability, cellular arrangement and morphology changes.

The substitution of alginate with its RGD-enriched variant significantly influenced cell development, as evidenced by microscopy images acquired after 7 and 21 days (Figure 5e). In samples with RGD-alginate treated with μTG, cells colonized the internal pores within 1 week, as expected. By 3 weeks, however, they had migrated to the external surfaces of the fibers, forming a uniform coating. When RGD-alginate scaffolds were not treated with μTG, the results differed dramatically, likely due to rapid degradation of both alginate and gelatin. This degradation can be attributed to the sequestration of Ca^2+^ ions, which are intercalated within the polymer strands by ion species present in the culture medium, as previously documented.^53^ As observed previously for C1, for untreated alginate (without RGD modification), cells largely remained within the fibers, occupying the voids created by air bubbles, and the effect of μTG did not show a clear outcome (Supporting Information, Figure S4b). This study highlights the combined benefits of RGD functionalization and enzymatic stabilization for promoting cell migration, adhesion, and scaffold integrity over extended culture periods.

In summary, the following conclusions regarding cellular activity within 3D-printed foams can be drawn. The presence of surfactant influenced cellular behavior in all conditions, even at very low concentrations, underscoring the importance of fine-tuning surfactant levels to balance foaming ability and cell viability. In scaffolds made from unmodified alginate, cells tended to migrate from the bulk material into the fibers’ internal pores, leading to spherical clusters within individual bubbles or elongated clusters in interconnected pores. The addition of RGD motifs to alginate facilitated cell migration out of the bulk material to the external surfaces of the fibers, resulting in a quasi-2D arrangement around the fibers. This effect was only observed in scaffolds treated with μTG, which enhanced mechanical stability and slowed material degradation. Without μTG, rapid scaffold degradation disrupted cell adhesion and viability. These preliminary studies demonstrate the feasibility of combining functionalized biomaterials and 3D bioprinting for creating advanced cell-laden scaffolds with tailored properties. They thus provide a foundation for designing viable 3D-bioprinted cell-laden foams. Further research will be necessary to understand the complex mechanisms underlying cell development in response to different biochemical and morphological conditions.

## 4 Conclusions

This work presents a novel method for the one-step biofabrication of functionally graded porous hydrogels incorporating living cells. The engineered microfluidic printing head enabled the production of microbubbles ranging from 30 to 70 μm, at frequencies up to 2 kHz, with bubble size and volume adjustable through modulation of air pressure and biomaterial ink flow rate. After bubble generation, the foam was converted into gel fibers and deposited layer-by-layer using a 3D printer according to a predetermined design.

We directly monitored the foaming process inside the microfluidic chip under various experimental conditions to establish calibration curves correlating bubble size and air volume fraction with gas pressure and liquid flow rate. A post-printing morphological analysis of the gel constructs was performed using μCT to characterize the porous texture in terms of pore diameter, pore volume, and spatial distribution. One challenge was the interfiber space created during the layer-by-layer deposition, which resulted in black areas that were difficult to distinguish from actual pores. To improve this, future work will focus on enhancing radiographic contrast^54^ and refining image processing techniques for better automation in pore detection.

The combined foaming and bioprinting approach developed in this study offers several advantages over other methods described in recent literature. First, it eliminates the need for organic solvents at any stage of the manufacturing process, making it ideal for cell incorporation in a bioprinting context. Second, the online gelation of the foam immediately after production prevents destabilization, maintaining printing fidelity and quality throughout extended printing periods. Integrating the microfluidic foam generator and coaxial extruder within a 3D printer allows precise control over foam characteristics, such as bubble size and foam density, and their translation into porous solids with predesigned profiles.

A further benefit is decoupling the foaming chip from the coaxial extruder, which helps preserve cell viability. By introducing cells into the foam after its formation, this setup avoids subjecting the cells to excessive shear stress, a common issue when cells are suspended directly in the biopolymeric solution before entering the foaming chip. This strategic separation preserves cellular integrity and ensures optimal foamability, balancing structural quality and biological performance.

However, a limitation of this method is the need for a surfactant, Plantacare 2000, which was found to be cytotoxic at the initial concentration. To mitigate this, foam dilution was required to reduce the surfactant concentration to a non-cytotoxic level, limiting the flexibility of tuning the material’s porous structure. This limitation could be overcome using a biocompatible surfactant derived from natural sources. Promising candidates include albumin^55^ and β-lactoglobulin.^56^ Albumin, a plasma protein, has a high absorption rate at the air/water interface and can suppress cytotoxic effects associated with conventional surfactants or, if sufficiently surface-active, may eliminate the need for any additional surfactants.^57^ Similarly, β-lactoglobulin fibrils have potential as a surface-active component, capable of forming adsorption layers at the air/water interface. Incorporation of a biocompatible surfactant could help maintain the integrity of the foam by preventing excessive dilution, thereby preserving the intended pore volumes and sizes. This approach would enable more systematic investigations into the intricate relationships between foam architecture, bioink composition, and cell maturation, facilitating more accurate tissue modelling and enhancing the reproducibility of experimental outcomes.

## Data Availability

Data will be available upon reasonable request from the authors.

## Supporting Information Available

Video S1: Time lapse of the 3D printing process of foamed and non-foamed constructs (MP4)
Video S2: 3D image of calcein-stained cells encapsulated in a non-foamed fiber on day 21 (MP4)
Video S3: 3D image of calcein-stained cells encapsulated in a foamed fiber on day 21 (MP4)
Additional experimental details, including photographs of 3D printed constructs, additional mechanical characterization, 3D reconstructions of stained cells in foamed and non-foamed fibers, and comprehensive results of cell viability studies (PDF)
