# Supplementary material for: Microfluidic 3D Bioprinting of Foamed Fibers with Controlled Micromorphology
Source: ACS Appl Mater Interfaces. 2025 Feb 18;17(9):13632–45. doi: 10.1021/acsami.4c22450 (PMC11891826; doi:10.1021/acsami.4c22450)
Supplement: Supplementary file 4 — am4c22450_si_004.pdf [file am4c22450_si_004.pdf]

Supporting Information

Microfluidic 3D bioprinting of foamed fibers with controlled micro-morphology

Federico Serpe[1,2], Francesco Nalin[3], Maria Celeste Tirelli[3], Pasquale Posabella[4], Nehar Celikkin[3], Jakub Jaroszewicz[4], Wojciech Święszkowski[4], Andrea Barbetta[1], Efsun Şentürk[2,5], Carlo Massimo Casciola[5], Giancarlo Ruocco[2], Gianluca Cidonio[2,5*], Chiara Scognamiglio[2*], Marco Costantini[3*]

[1] Department of Chemistry, University of Rome "La Sapienza", 00185 Rome, Italy
[2] Center for Life Nano- & Neuro- Science – CLN2S, Italian Institute of Technology (IIT), 00161 Rome, Italy
[3] Institute of Physical Chemistry – Polish Academy of Sciences, 01224 Warsaw, Poland
[4] Warsaw University of Technology, Faculty of Materials Science and Engineering, 02507 Warsaw, Poland
[5] Department of Mechanical and Aerospace Engineering (DIMA), University of Rome "La Sapienza", 00184 Rome, Italy

* Corresponding Authors:

Prof. Marco Costantini, mcostantini@ichf.edu.pl
Dr Chiara Scognamiglio, chiara.scognamiglio@iit.it
Dr Gianluca Cidonio, gianluca.cidonio@uniroma1.it

Polydispersity index (PDI) calculation

$$PDI\ (\%) = \frac{\sqrt{\langle d_{bubble}^2 \rangle - \langle d_{bubble} \rangle^2}}{\langle d_{bubble} \rangle} \cdot 100 \qquad (S1)$$

Equation S1 – Formula for the calculation of the PDI.

Air pressure (mBar)	PDI (%)
700	2.69
800	8.50
900	6.91
1000	5.09
1200	2.54
1400	2.58
1600	8.52

Table S1 – PDI of generated bubbles at different pressure conditions with fixed liquid flowrate of 35 µl/min.

Video S1 (attached) – Time lapse of the 3D printing process showing a comparison between simple printing and foam printing to realize 3D constructs with scalable dimensions and different shapes.

Video S2 (attached) – 3D reconstruction of calcein-stained cells encapsulated in a non-foamed fiber on day 21.

Video S3 (attached) – 3D reconstruction of calcein-stained cells encapsulated in a foamed fiber on day 21.

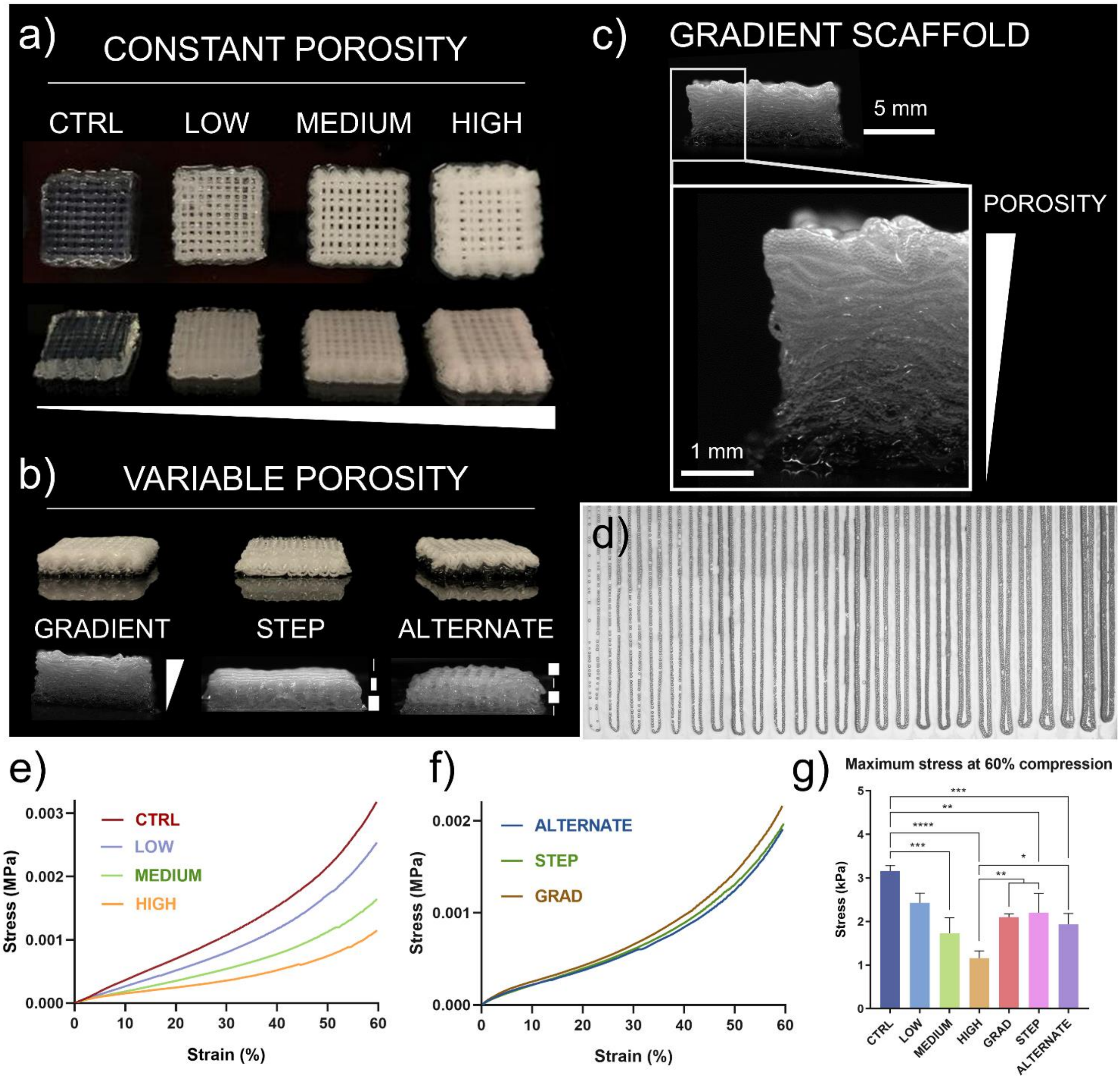

Figure S1 – 3D printing and mechanical compression tests of simple and composite scaffolds. a) Images of the samples tested with constant porosity. b) Images of the samples tested with variable porosity. c) Photograph of a 3D-printed porous scaffold with a gradual increase of porosity from the bottom to the top. d) Printing of a single filament while air pressure is constantly increased (from the bottom to the top. e) Stress-strain curves of the samples with constant porosity and the control scaffolds. f) Stress-strain curves of the samples with variable porosity. g) Maximum stress at 60% of compression of simple and composite 3D printed porous constructs. Statistical significance was calculated via one-way ANOVA. Results are expressed as mean ± SD of at least three replicates for each experiment, *p<0.05, **p<0.01, ***p<0.001, ****p<0.0001.

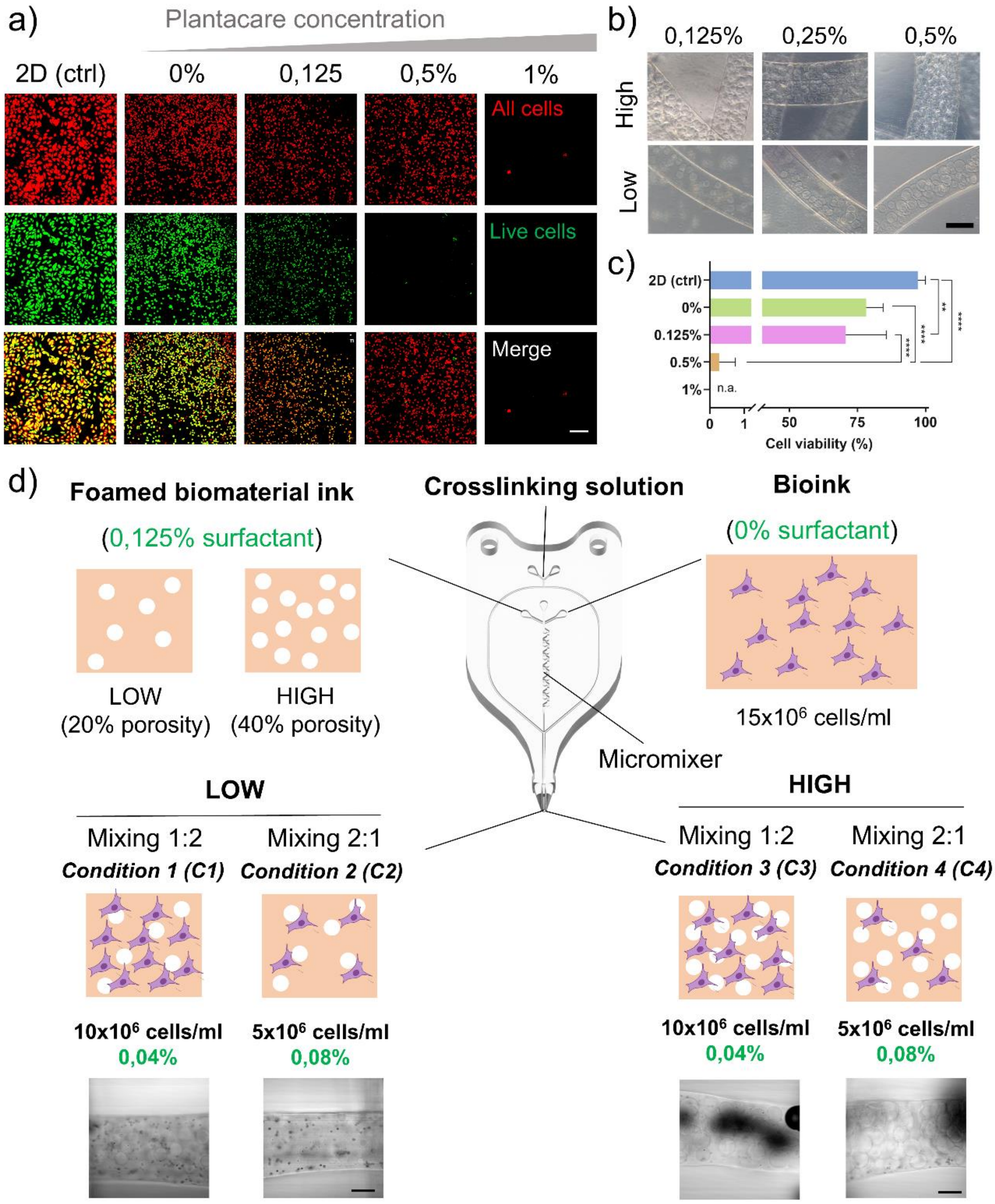

Figure S2 – Analysis of viability and description of preliminary cell experiments. a) Confocal images of cell viability at different surfactant (Plantacare 2000) concentrations. The scale bar is 200 µm. b) Images of the porous fibers obtained at different Plantacare concentrations with high and low porosity. The scale bar is 200 µm. c) Graph of cell viability at different Plantacare 2000 concentrations after 24 hours from encapsulation. d) Sketch representing the different experimental conditions of preliminary bioprinting experiments. For each condition it is reported a draft representing the quantity of porosity and cellular density, the eventual concentration of surfactant (in green), and a brightfield image of the cell-laden fiber. Scale bars are 100 µm. Statistical significance was calculated via one-way ANOVA. Results are expressed as mean ± SD of at least three replicates for each experiment, $^{**}p<0.01$, $^{****}p<0.0001$.

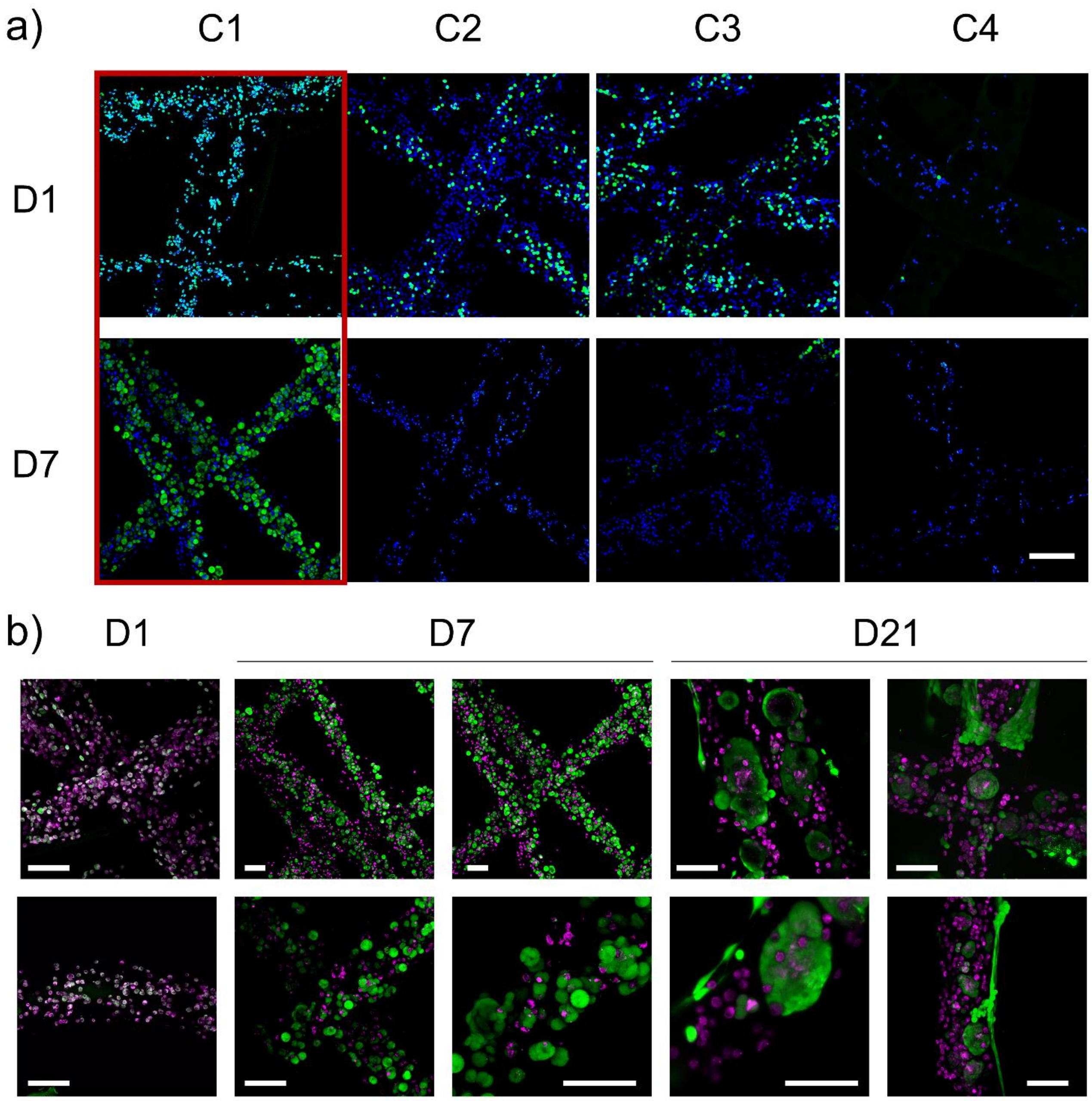

Figure S3 – Cell viability experiments. a) Viability assay performed on four experimental conditions C1, C2, C3 and C4 on day 1 and 7. The scale bar is 200 µm. b) Viability assay of condition 1 (C1) on days 1, 7, and 21. All scale bars are 200 µm.

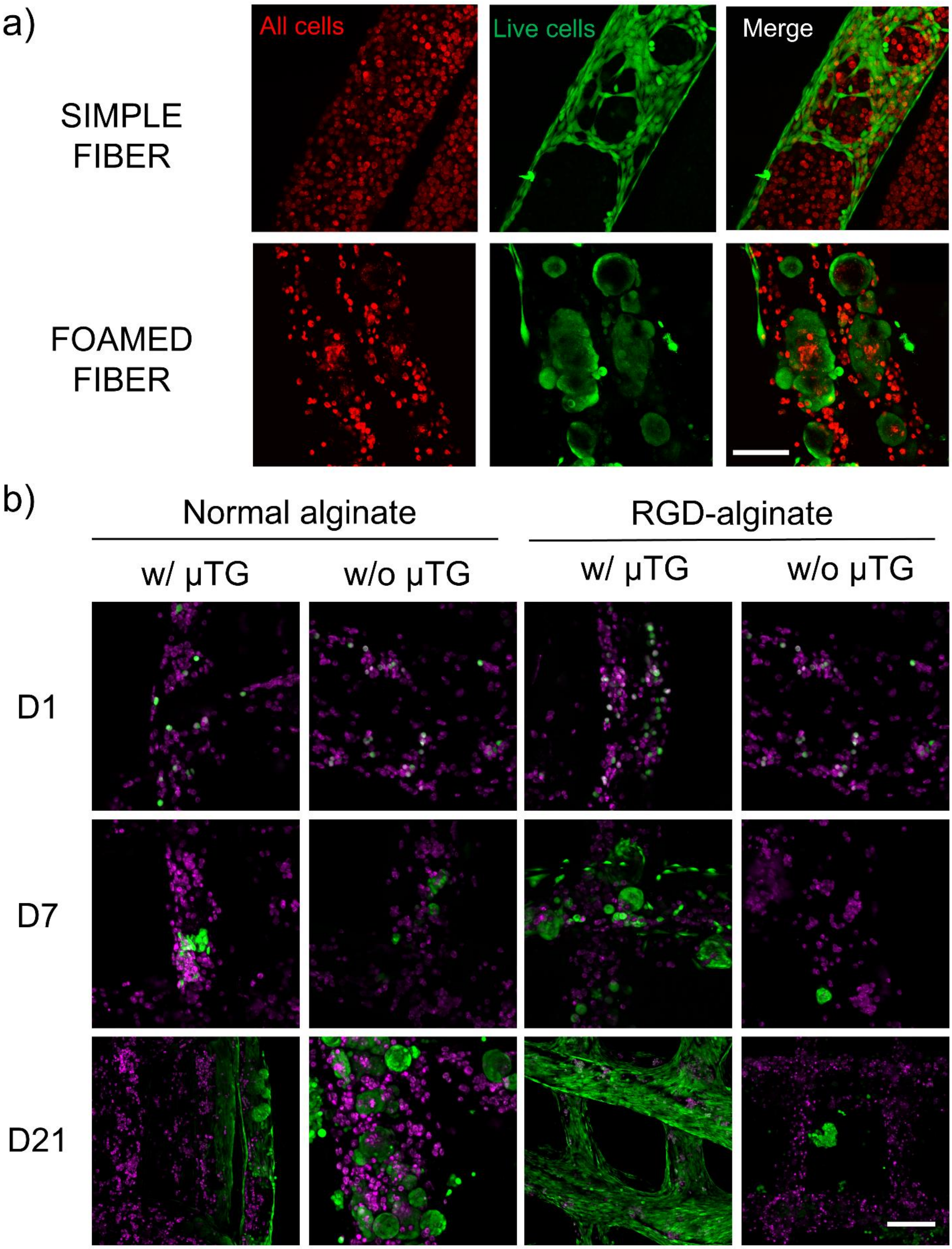

Figure S4 – Cell viability experiments. a) Comparison of the viability of cells printed in normal and foamed bioink on day 21. The scale bar is 100 µm. b) Viability of cells printed in the modified bioink with RGD-alginate and/or micro-transglutaminase (µTG) tested on days 1, 7, and 21. The scale bar is 200 µm.
